# Micronutrients Deficiencies in Candidates of Bariatric Surgery: Results from a Single Institution over a 1-Year Period

**DOI:** 10.1007/s11695-022-06355-8

**Published:** 2022-11-04

**Authors:** Giovanna Berardi, Antonio Vitiello, Adam Abu-Abeid, Vincenzo Schiavone, Antonio Franzese, Nunzio Velotti, Mario Musella

**Affiliations:** 1 Advanced Biomedical Sciences Department, Naples “Federico II” University, AOU “Federico II”—via S.Pansini, 80131 Naples, Italy; 2 Division of General Surgery, Affiliated to Sackler Faculty of Medicine, Tel Aviv Sourasky Medical Center, Tel Aviv University, 6 Weizman Street, 64230906 Tel Aviv, Israel

**Keywords:** Bariatric metabolic surgery, Severe obesity, Micronutrient deficiency, Vitamin deficiency

## Abstract

**Background:**

Micronutrient deficiencies represent a common condition after bariatric surgery (BS). The prevalence of these nutritional disorders before BS is still debated. The aim of our study was to retrospectively evaluate the prevalence of micronutrient deficiencies in candidates for BS.

**Methods:**

A prospectively maintained database of our institution was searched to find all patients who underwent surgery between January and December 2021. The following data were collected: age, gender, body mass index (BMI), obesity-associated diseases, and preoperative serum levels of vitamin B12, folate, and vitamin D.

**Results:**

A total of 174 patients were included in our study. Mean age and BMI were 39.2 ± 11.4 years and 44.3 ± 7.1 kg/m^2^, respectively. One hundred and thirty-nine patients (79.9%) had at least one preoperative micronutrient disorder, with vitamin D deficiency being the most common (116, 66.7%), followed by a deficit of folate (76, 43.7%) and vitamin B12 (10, 5.7%). Forty-seven (27%) individuals had insufficient levels of vitamin D. Comparison of deficiencies between sexes showed that vitamin B12 < 20 ng/ml was significantly more frequent in women (*p* = 0.03). DLP showed a mild significant effect on folate levels (*p* = 0.01), while the association of HNT and T2DM had a mild significant effect on vitamin B12 (*p* = 0.02).

**Conclusions:**

Preoperative micronutrient deficiencies were frequently found in candidates for BS. Approximately 90% of patients had deficient or insufficient serum levels of vitamin D preoperatively. Almost half of the patients had a preoperative deficit of folate, and vitamin B12 deficiency was significantly more frequent in the female population. It is mandatory to screen all patients undergoing BS for vitamin deficiencies before surgery.

## Introduction

The worldwide prevalence of severe obesity is continuously increasing. Currently, a third of the world’s population is considered overweight or obese [1–3].

BS represents the most effective treatment for patients with severe obesity, and it is associated with sustained weight loss, remission of obesity-related diseases, and reduced mortality [4, 5].

Even though severe obesity is often associated with excessive consumption of calories, nutritional deficiencies are rarely found in BS candidates [6, 7]. The most commonly reported are deficiencies of vitamin D, vitamin B12, folate, and iron [8]. Moreover, the restrictive and/or hypoabsorptive mechanisms of BS may induce postoperative de novo deficiency of thiamine, vitamins B12 and D, folate, iron, and copper. However, reports of severe clinical manifestations have been sporadically described [9–14].

The purpose of this study was to determine the prevalence of micronutrient deficiencies in our patients before BS. We anticipated that the prevalence of micronutrient deficiencies to be high in patients with severe obesity as these patients can mistakenly be considered to not have malnutrition.

## Methods

This was a cross-sectional study based on a prospectively collected database of a single bariatric center. All patients submitted to BS from January 1 to December 31, 2021, at our institution were included in the present research. All participants signed informed consent.

All candidates of BS underwent preoperative multidisciplinary evaluation and met the American Society of Metabolic and Bariatric Surgery (ASMBS) guidelines.

The following baseline demographic and clinical characteristics were retrieved from our database: age, gender, BMI, sex (M/F), obesity-related diseases, preoperative serum levels of vitamin B12, folate, and vitamin D. All individuals with prior history of bariatric surgery were excluded from the study population.

### Definition of Micronutrient Deficiency

Micronutrients deficiencies were defined in accordance with the most recent published guidelines of the ASMBS [15]:

- Vitamin B12: < 200 pg/ml deficiency, < 400 pg/ml suboptimal
- Folate: < 3.4 ng/ml deficiency
- Vitamin D: 20–30 ng/mL insufficiency, < 20 ng/ml deficiency

### Statistical Analysis

Statistical analysis was performed using IBM SPSS version 28. Continuous data were expressed as mean values ± standard deviation, while frequencies were reported as percentages. Shapiro–Wilk test was performed to test normality; ANOVA test and chi-square test (or Fisher’s test when appropriate) were respectively used to compare means and percentages. Correlations among variables were measured using the Pearson correlation coefficient, while a general multivariate regression model was used to analyze the effects of preoperative obesity-related diseases (HTN, T2DM, and DLP) on serum levels of vitamins. All *p*-values were considered significant when < 0.05.

## Results

### Study Participants

A total of 214 patients underwent BS during the study period. Data were not available for 26 individuals, while 14 were excluded due to the previous BS; thus, 174 patients undergoing BS were eventually included in our analysis.

Eighty-two (46.9%) patients underwent sleeve gastrectomy (SG), while 83 (48%) were submitted to the one anastomosis gastric bypass (OAGB), and 8 (4.6%) to the Roux-en-Y gastric bypass.

One hundred and twenty-seven (73%) patients undergoing BS were women. Mean age and BMI were 39.2 ± 11.4 years and 44.3 ± 7.1 kg/m^2^, respectively. Baseline anthropometric and nutritional characteristics of the study population are depicted in Table 1; no statistical difference was found between male and female patients.

**Table 1:** Medical and anthropometric characteristics of MBS candidates according to sex

		*N*	Mean ± standard deviation	*P*-value
BMI (kg/m^2^)	*Female*	127	43.8 ± 6.6	0.185
	*Male*	47	45.5 ± 8.2	
	*Total*	174	44.3 ± 7.1	
Age (years)	*Female*	127	38.9 ± 11.5	0.583
	*Male*	47	39.9 ± 11.1	
	*Total*	174	39.2 ± 11,4	
Vit_B12 (pg/ml)	*Female*	127	344.8 ± 111.7	0.980
	*Male*	47	344.4 ± 96.1	
	*Total*	174	344.7 ± 107.4	
Folate (ng/ml)	*Female*	127	4 ± 2.3	0.827
	*Male*	47	4.1 ± 2.4	
	*Total*	174	7.7 ± 34.8	
Vit_D (ng/ml)	*Female*	127	16.9 ± 8.2	0.635
	*Male*	47	17.6 ± 7.7	
	*Total*	174	17.1 ± 8	

Rates of patients with type 2 diabetes mellitus (TD2M) or dyslipidemia (DLP) were comparable between the sexes, while hypertension (HTN) was statistically more frequent in the male population (Table 2).

**Table 2:** Rate of patients suffering from type 2 diabetes mellitus (TD2M), hypertension (HTN), and dyslipidemia (DLP) according to sex

		Sex *n* (%)		Total *n* (%)	*P*-value
		F	M		
TD2M	No	120 (94.5)	41 (87.2)	161 (92.5)	0.1062
	Yes	7 (5.5%)	6 (12.8)	12 (7.5%)	
	Total			174	
HTN	No	101 (79.5)	23 (48.9)	124 (71.3)	0.000*
	Yes	26 (20.5)	24 (51.1)	50 (21.7)	
	Total			174	
DLP	No	120 (94.5)	41 (87.2)	161 (92.5)	0.1062
	Yes	7 (5.5%)	6 (12.8)	12 (7.5%)	
	Total			174	

^*^ = *p* < 0.05

### Micronutrient Status

One hundred and thirty-nine patients (79.9%) had at least one micronutrient disorder, with vitamin D deficiency being the most common (116, 66.7%), followed by a deficit of folate (76, 43.7%) and vitamin B12 (10, 5.7%). Forty-seven (27%) individuals had insufficient serum levels of vitamin D. Comparison of deficiencies between sexes showed that vitamin B12 < 20 ng/ml was significantly more frequent in women (Table 3).

**Table 3:** Comparison of vitamins deficiencies between sexes

			Sex		Total	*P*-value
			Female	Male		
Vit. B12 deficiency	No	*N*	117	47	164	0.03*
		%	92.1%	100.0%	94.3%	
	Yes	*N*	10	0	10	
		%	7.9%	0.0%	5.7%	
Total			127	47	174	
Folate deficiency	No	*N*	74	24	98	0.25
		%	58.3%	51.1%	56.3%	
	Yes	*N*	53	23	76	
		%	41.7%	48.9%	43.7%	
Total			127	47	174	
Vit. D deficiency	No	*N*	42	16	58	0.52
		%	33.1%	34.0%	33.3%	
	Yes	*N*	85	31	116	
		%	66.9%	66.0%	66.7%	
Total			127	47	174	

boldface means^*^p < 0.05

A significant correlation between serum levels of folate and vitamin B12 and between age and serum levels of folate was found (Table 4).

**Table 4:** Correlation between baseline age, BMI, and serum levels of vitamin D, B12, and folate

		BMI	Vit_B12	Folate	Vit_D	Age
BMI	Pearson’s correlation	1	− 0.013	− 0.098	− 0.044	− 0.040
	Sign. (two-tailed)		0.867	0.200	0.568	0.602
	*N*	174	174	174	174	174
Vit_B12	Pearson’s correlation	− 0.013	1	0.250	0.141	0.043
	Sign. (two-tailed)	0.867		0.001	0.064	0.572
	*N*	174	174	174	174	174
Folate	Pearson’s correlation	− 0.098	0.250	1	− 0.001	0.251
	Sign. (two-tailed)	0.200	**0.001**		0.992	**0.001**
	*N*	174	174	174	174	174
Vit_D	Pearson’s correlation	− 0.044	0.141	− 0.001	1	0.110
	Sign. (two-tailed)	0.568	0.064	0.992		0.149
	*N*	174	174	174	174	174
Age	Pearson’s correlation	− 0.040	0.043	0.251	0.110	1
	Sign. (two-tailed)	0.602	0.572	**0.001**	0.149	
	*N*	174	174	174	174	174

boldface means *p* < 0.05

DLP showed a mild significant effect on folate levels, while the association of HNT and T2DM had a mild significant effect on vitamin B12. No other significant effect of preoperative obesity-related diseases on vitamins level was found (Table 5).

**Table 5:** General multivariate regression model of the effects of preoperative obesity related diseases (*HTN*, hypertension; *T2DM*, type II diabetes mellitus; *DLP*, dyslipidemia) on serum levels of vitamins

Between subjects test						
Independent variable	Dependent variable	Quadratic sum	df	Quadratic mean	*F*	Sig.
TD2M	Vit_B12	18,792,779	1	18,792,779	1.692	0.195
	Folate	0.525	1	0.525	0.100	0.752
	Vit_D	8640	1	8640	0.134	0.715
HTN	Vit_B12	1437,016	1	1437,016	0.129	0.720
	Folate	1689	1	1689	0.322	0.571
	Vit_D	28,485	1	28,485	0.442	0.507
DLP	Vit_B12	2,724,461	1	2,724,461	0.245	0.621
	Folate	30,043	1	30,043	5.727	0.018*
	Vit_D	73,255	1	73,255	1.136	0.288
TD2M * HTN	Vit_B12	53,612,370	1	53,612,370	4.827	0.029*
	Folate	2459	1	2459	0.469	0.495
	Vit_D	129,913	1	129,913	2.014	0.158
TD2M * DLP	Vit_B12	4,326,903	1	4,326,903	0.390	0.533
	Folate	6778	1	6778	1.292	0.257
	Vit_D	1807	1	1807	0.028	0.867
HTN * DLP	Vit_B12	17,398,947	1	17,398,947	1.566	0.212
	Folate	7498	1	7498	1.429	0.234
	Vit_D	31,157	1	31,157	0.483	0.488

^*^ = *p* < 0.05

## Discussion

Primary and secondary BS represents the best treatment for patients with severe obesity [16–18]. However, BS has been associated with nutritional deficiencies in several studies in both restrictive and malabsorptive procedures [19]. Paradoxically, when evaluating patients with severe obesity for BS, it is common to encounter nutritional deficiency preoperatively [7, 8, 20].

The reasons for micronutrient deficiency in severe obesity are not fully understood. In a recent review [21], several mechanisms have been proposed, such as overconsumption of foods with high calories but low nutrient densities, diets with a high-fat content associated with lower intake of vitamins, increased storage of vitamin D in adipose tissue in patients with increased body fat, and possible relation to microbiota dysfunction.

Some authors have also correlated malnutrition to low levels of newly discovered hormones like fibroblast growth factor 23 (FGF 23) [22]. Noteworthy, bariatric surgery induces changes in the circulating concentrations of FGFs [23, 24].

Rates of at least one micronutrient deficiency prior to BS are reported to be 80–90%, with vitamin D deficiency being the most commonly reported deficient micronutrient [8, 20, 21]. Similarly, our study showed that vitamin D deficiency was the most commonly deficient micronutrient occurring in 66.7% of the study population. In addition, we showed that 27% of the patients had vitamin D insufficiency. Therefore, approximately 90% of patients had deficient or insufficient serum levels of vitamin D preoperatively. Vitamin D deficiency has been shown to be associated with several pathologic conditions—osteoporosis, muscle atrophy, recurrent falls, as well as cancer, TD2M, and increased respiratory infections [25]. Its impact can cause severe morbidity and, therefore, it is of major importance to treat it accordingly. Moreover, other micronutrient deficiencies can be associated with severe morbidity in patients with severe obesity. Folate deficiency was shown to occur in 43.7% of our cohort. Interestingly, folate deficiency has been linked with the development of cardiovascular diseases, anemia, and birth defects and may cause neuropsychiatric manifestations [26]. Interestingly, it is reported to be associated with higher BMI, higher body fat accumulation, and greater waist circumference [27]. This further supports the importance of treating folate deficiency in patients with severe obesity. Vitamin B12 deficiency was diagnosed in a relatively low prevalence of our cohort (5.7%), it may be associated with several negative consequences if untreated, such as megaloblastic anemia, glossitis, and sub-acute degeneration of the spinal cord [28]. We strongly recommend evaluating levels of both folate and vitamin B12 before initiating treatment in patients with severe obesity, as treatment of folate deficiency may improve the patient’s symptoms and mask an underlying vitamin B12 deficiency. We could not address iron deficiency in our cohort due to lack of data. However, it is important to note that its prevalence may be high in patients with severe obesity as it was shown that severe obesity disrupts iron homeostasis and weight reduction can notably reduce the release of inflammatory cytokines and hepcidin, which increases iron absorption [29].

Micronutrient deficiency prior to BS was shown to be higher in women than in men [30–32], particularly vitamin D and iron deficiency [8]. Vitamin B12 deficiency was shown to occur in 10.6% of women undergoing BS [33]. Interestingly, vitamin D deficiency was reported to be as high as 6% in women of childbearing age despite adequate intake [34], and vitamin B12 deficiency was shown to strongly correlate with overweight and obesity [35]. Among our BS candidates, the deficit of vit. B12 was more frequent in women and occurred in 10 patients (5.7%), all of which were women. However, other published articles did not show a significant difference in micronutrient deficiency levels when comparing women and men [36, 37]. Other baseline characteristics, such as age and obesity-related diseases, did not significantly influence the serum levels of the studied vitamins.

In the last decade, several guidelines have been published regarding preoperative nutritional care for patients undergoing BS [38–40]. However, BS in patients who suffer from preoperative nutritional deficiencies may worsen these disorders and it may also induce de novo deficits [41].

The type of procedure performed may play a critical role in the development of micronutrient deficiency postoperatively. Krzizek et al. [42] have reported RYGB to have significantly higher deficits when compared to SG in 1-year follow-up in levels of hemoglobin (36.7% vs 18.4%), ferritin (32.4% vs 9.3%), vitamin B12 (16.7% vs 4.9%), vitamin D (90.3% vs 49.2%), vitamin A (33.3% vs 9.0%), and selenium (66.7% vs 6.1%). At 3 years of follow-up, the differences persisted for hemoglobin, ferritin, and vitamin D. OAGB has gained popularity in the last decade and is considered one of the most commonly performed metabolic bariatric procedures worldwide [43]. OAGB has been reported to have more nutritional deficiencies when compared to RYGB [44, 45], and in a survey of metabolic bariatric surgeons which aimed to understand the objection to OAGB, malnutrition emerged as one of the major concerns to not performing OAGB [46]. However, in the recently published multi-institutional survey regarding revisional surgery after OAGB by Musella et al. [47], only 0.13% of patients underwent OAGB revision due to excess weight loss and malnutrition. Carbajo et al. reported that 20% of patients require long-term vitamin D supplementation after OAGB, and 2% of OAGB patients requiring long-term vitamin B12 and folate supplementation [48]. A recent article has also demonstrated that postoperative CONUT score and micronutrient deficiency were comparable after RYGB and OAGB [49]. Even if rarely reported, serious nutritional deficiencies may occur also after LSG [50].

Our study has several limitations. It is of retrospective nature, with no comparative group of patients with normal weight, and we did not address all micronutrients. Moreover, differences between pre- and postmenopausal women could not be analyzed. Despite that, we collected the data prospectively, the cohort is relatively large compared to men versus women, and all micronutrient deficiencies were defined according to recent guidelines.

## Conclusion

Preoperative micronutrient deficiencies were frequently found in patients undergoing BS. Approximately 90% of patients had serum levels of vitamin D below the normal threshold preoperatively, while vitamin B12 deficiency was significantly more frequent in the female population. Age and obesity-related diseases did not significantly influence the serum level of the studied nutrients. Due to the risk of postoperative worsening of these nutritional disorders, it is mandatory to screen all candidates of BS and treat them accordingly before surgery.
